# Symbiotic anti-oxidant, anti-glycation, and anti-inflammatory qualities of a combination of thiamine and niacin protected type-2 diabetic male rats against both macro and micro-vascular complications

**DOI:** 10.22038/ijbms.2024.77553.16771

**Published:** 2025

**Authors:** Sina Mahdavifard, Hamid Reza Malekzadeh

**Affiliations:** 1 Department of Clinical Biochemistry, Faculty of Medical Sciences, Ardabil University of Medical Sciences, Ardabil, Iran; 2 Faculty of Medical Sciences, Ardabil, Iran

**Keywords:** Diabetes complications, Glycation, Glyoxalase-I, Niacin, NF-kappa β, Oxidative stress, Thiamine

## Abstract

**Objective(s):**

Increased nuclear factor (NF-kβ) and carbonyl stress due to decreased glyoxalase-1 activity (Glo-I) contribute significantly to insulin resistance and vascular complications. Therefore, we aimed to study the impact of the combination of thiamine and niacin on hepatic NF-kβ signaling, metabolic profile, and Glo-I activity in male rats with type-2 diabetes (T2DM).

**Materials and Methods:**

Forty male rats were divided equally into five groups: control, diabetic, diabetic treated with thiamine (180 mg/l in drinking water), niacin (180 mg/l), and a combination of both. The treated groups received the treatments daily in drinking water for two months. T2DM was induced using a combination of nicotinamide and alloxan. Metabolic profile and renal dysfunction parameters were assessed. Additionally, various glycation, oxidative stress, and inflammatory markers were measured.

**Results:**

The treated group with both vitamins showed the lowest blood sugar and insulin resistance indices, cardiovascular indices, renal dysfunction parameters, hepatic NF-kβ expression, oxidative stress, inflammatory and glycation markers, and the highest anti-oxidant and anti-glycation markers, β cell activity, and insulin sensitivity. Thiamine exhibited more anti-inflammatory activity than niacin in diabetic rats, while niacin demonstrated stronger anti-oxidant activity (*P*<0.001).

**Conclusion:**

The combined use of vitamins had a more beneficial impact on macro and microvascular complications in diabetes than each alone, attributed to their higher anti-oxidant, anti-inflammatory, and anti-glycation characteristics. The vitamins also had a more corrective effect on glucose-lipid metabolism, insulin sensitivity, and renal function through a stronger lowering effect on hepatic NF-kβ expression.

## Introduction

Insulin resistance is the main factor contributing to vascular complications as the principal cause of death worldwide (1). The increase in hepatic nuclear factor (NF-kβ) (2) and carbonyl stress (methylglyoxal accumulation) due to decreased activity of glyoxalase-1 (Glo-I) are key (3) contributors to insulin resistance, high blood glucose, dyslipidemia, diabetes, and its vascular complications. Glo-I plays a crucial role in protecting against insulin resistance, metabolic syndrome, and diabetes vascular complications by detoxifying MGO (4). Therefore, *increasing* Glo-I activity and reducing NF-kβ (2) are important preventive and therapeutic strategies for diabetes complications (5). 

Thiamine and niacin have anti-oxidant properties and play a role in energy production (6). Thiamine has a vital role in metabolism and insulin function (7). Probably, thiamine shortage, as a common feature in type 2 diabetes, has a cardinal role in insulin resistance and dyslipidemia (8). Thiamine, with its beneficial properties including anti-oxidant, anti-glycation, and anti-inflammatory effects, improves diabetes vascular complications (9). Niacin has benefits for dyslipidemia (10), glycemia, oxidative stress, and DNA damage in diabetic patients and models (11). Building on these studies, a new combination treatment was developed to explore more useful treatments by combining the effects of thiamine and niacin to prevent or improve diabetes vascular complications. To investigate the impact of these vitamins and their combination, a study was conducted on type 2 diabetic rats to assess their effects on the NF-kβ expression, metabolic profile, and Glo-I activity. The study also examined the impact of the treatments on glycation and oxidation of albumin (Alb) and LDL. 

## Materials and Methods

***Materials***

Alloxan, chloramine T, thiobarbituric acid, acetonitrile, guaiacol, H_2_O_2_, ketamine and xylazine, paraoxon, methylglyoxal, and phosphate buffer components were purchased from the Merck Company.

***Type 2 diabetes induction***

Male Wistar rats (190±15 g weight and seven weeks old) were obtained from the Pasteur Institute, Karaj, Iran. The animals were housed in standard conditions. The project was authorized by the Ethics Committee of Ardabil University of Medical Sciences (IR.ARUMS.REC.1401.30). Type 2 diabetes mellitus (T2DM) was induced in rats using a combination of nicotinamide and alloxan (120+ 50 mg/kg) (12). After seven days, rats with fasting blood sugar (FBG) levels between 8–16 mmol/l and postprandial blood sugar levels > 11 mmol/l were confirmed as T2DM rats. Additionally, the induction of T2DM was validated by calculating the Homeostasis Model Assessment of Insulin Resistance (HOMA-IR). The percentage of beta-cell activity (%B) and insulin sensitivity (%S) were also calculated using the HOMA2 calculator software.

Forty rats were divided into five equal groups: control (C) and diabetic (D). The diabetic rats received 180 mg/l of thiamine, 180 mg/l of niacin, or a combination of both in their drinking water, labeled as D (Thiamine), D (Niacin), and D (Thiamine & Niacin), respectively. The treated groups received the daily treatment for two months, following dosages and administration methods outlined in the literature (13). The treatments were added to the drinking water to avoid the stress induced by gavage or intraperitoneal (IP) injection. The rats′ body weight was measured one week before the end of the study. 24-hour urine samples were collected. After 16 hr of fasting and desensitization with an IP injection of ketamine and xylazine, blood samples were taken from the rat′s heart and placed into test tubes. The rats were euthanized immediately after blood collection, and their livers and kidneys were promptly separated and weighed.

***Determination of biochemical parameters***

FBG, triglycerides (TG), total cholesterol (TC), LDL, HDL, creatinine (Cr), and urine protein excretion (PU) were assessed using commercial kits. The glomerular filtration rate (GFR) was calculated using an equation (Eq.1). 

$\mathrm{GFR}=\frac{\mathrm{Urine creatinine}}{\mathrm{Serum Creatinine}}\times \frac{\mathrm{Urine volume}}{\mathrm{Bodyweight}}$ Eq. 1

Insulin levels were determined with an ELISA) kit (ZellBio GmBH, Germany). Furthermore, the homeostasis model assessment of insulin resistance (HOMA-IR) was estimated using equation 2 (Eq.2). 

$\mathrm{HOMA}-\mathrm{IR}=\frac{\mathrm{FBS}\left(\frac{\mathrm{mmol}}{1}\right)\times \mathrm{Insulin}(\frac{\mathrm{\mu U}}{\mathrm{ml}})}{22.5}$ Eq. 2

Furthermore, beta-cell activity (%B) and insulin sensitivity (%S) were estimated using the HOMA2 calculator software (14). 

***Measuring glycated products and Glo-I activity***

Glycated albumin (g-Alb) was detected by measuring the reduction of tetrazolium chloride (NBT) and reading the absorbance at 530 nm (15). Glycated LDL (g-LDL) was determined by reacting extracted LDL with oxalic acid and thiobarbituric acid to produce a chromogen, the absorbance of which was measured at 443 nm. MGO was assessed using HPLC-UV(16). AGEs were measured with a fluorimeter at the emission maximum of 440 nm. Glo-I activity was evaluated by measuring the initial rate of formation of S-D-lactoylglutathione at 240 nm. 

***Quantifying oxidative stress and inflammatory markers***

LThe level of malondialdehyde (MDA) in the serum was quantified by measuring the absorbance of thiobarbituric acid derivatives at 535 nm. Advanced oxidation protein products (AOPP) were measured by detecting the absorbance at a wavelength of 340 nm in diluted serum (17). Primary LDL oxidation products in cyclohexane were measured at 234 nm. The final products of LDL oxidation were measured using a fluorimeter at 430 nm with excitation at 360 nm. Liquid chromatography was used to measure reduced glutathione (GSH) at 210 nm and sodium perchlorate at 100 mmol/l as a mobile phase (18). Paraoxonase-1 (PON1) activity was determined by measuring p-nitrophenol absorbance at 412 nm for one minute. Catalase (CAT) activity was measured using a modified Abi method. 

The inflammatory markers as interleukin-1β (IL-1β) and transforming growth factor-β (TGF-β1) were quantified with ELISA kits (Immunotech, France). The sera myeloperoxidase (MPO) activity was measured by detecting the absorbance of oxidized guaiacol at 470 nm. 

***Determination of NF-κB expression***

RNA was extracted from the rat liver, and the quantity and purity of RNA were determined. Quantitative real-time PCR (qRT-PCR) was carried out using a perfect SYBR-Green PCR kit (Toyobo, Japan). β-actin (ACTB) serves as the internal control for expression normalization. The primer sequences used for RT-PCR were: 

NF-kβ: 5´-CCTGTCTGCACCTGTTCCAA-3´ (forward), 3´ACTCCTGGGTCTGTGTTGTT-5´(reverse) ACTB: 5´-GGAGAA GATTTGGCACCACACT-3´ (forward) and 3´-CGGTTGGCCTTAGGGTTCAGA-5´ (reverse). 

Following normalization, the 2^- ΔΔCT^ method was selected to express relative expression levels CT (ACTB).

***Tube study***

*Determination of the effect of the vitamins on the formation of glycation and oxidation products of albumin and LDL*

Alb and LDL were extracted from the serum of rats incubated with and without thiamine (180 mg/l), niacin (180 mg/l), and their combination. 

*Determination of gly-oxidation products*

The glycation and oxidation products of Alb and LDL were detected using the procedures outlined in section 2.2.2.

***Statistical assessment***

Various parameters in the groups were evaluated by the multiple analysis of variance (MANOVA-TUKEY) tests in SPSS version 16.

## Results

***Animal study***

**Figure 1:**
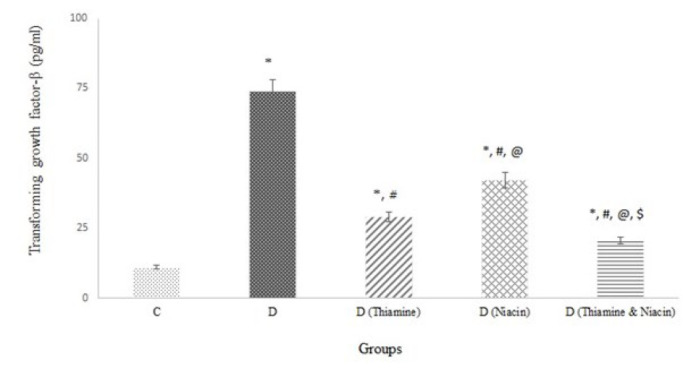
Transforming growth factor-β (TGF-β) levels in all rat groups

Table 1 displays metabolic clusters (FBS, insulin, HOMA1, HOMA2, B%, S%, serum lipids, and LDL/HDL ratio), kidney dysfunction markers, and body weight levels in rats treated and untreated with thiamine and niacin alone and in combination. Blood sugar, insulin, HOMA, and body weight levels were higher, and %B and %S in the untreated diabetic group were lower than in the treated ones. The lowest FBS, insulin resistance indices, body weight, and the highest %B and %S were observed in the group receiving both thiamine and niacin. Additionally, D (Thiamine) and D (Niacin) ranked second and third in all cited parameters (*P*<0.001). There was no difference in body weight between D (Thiamine) and D (Niacin). The lipid profile and LDL/HDL ratio in the treated ones were less than in the untreated ones. D (Thiamine & Niacin) group had the lowest TC, LDL, TG, FFAs, and LDL/HDL ratio. There was no difference in lipid profile between D (Thiamine) and D (Niacin). Still, the MPO/HDL, MPO/HDL, and MPO/PON-1 ratios in thiamine-treated diabetic rats were lower than in niacin-treated rats. The diabetic group had the highest Cr, PU, KWI (Table 1), TGF-β (Figure 1), and the lowest GFR quantities (Table 1). The cited parameters in D (niacin) were higher than in D (Thiamine). Furthermore, D (Thiamine & Niacin) had the lowest Cr, PU, TGF-β, and KWI levels and the highest GFR (*P*<0.001*).  *

**Figure 2:**
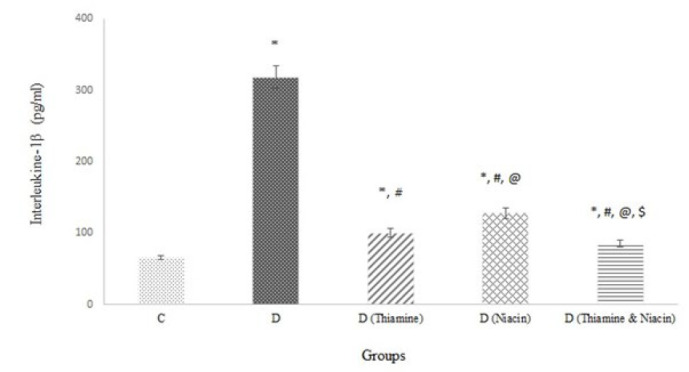
Interleukine-1β (IL-1β) level in control (C) and untreated diabetic (D) and treated diabetic rats

Table 2 and Figure 2 represent markers of oxidative stress (MDA, AOPP, and LDL oxidation products), inflammation (IL-1β and MPO), glycation, anti-glycation (Glo-I activity), and anti-oxidant (CAT, PON-1, and GSH). Untreated D rats exhibited the highest levels of oxidative stress, inflammation, and glycation indices, while anti-glycation and anti-oxidant markers were the lowest. The lowest levels of oxidative stress, inflammation, and glycation markers, along with the highest levels of anti-glycation and anti-oxidant markers, were observed in D (thiamine and niacin). Oxidative stress and anti-oxidant markers were lower and higher, respectively, in D (Thiamine) than in D (Niacin) and vice versa for inflammatory markers. Early glycation products (g-Alb and g-LDL), intermediate glycation products (MGO), and end glycation products (AGEs) levels were highest in the D group compared to treated groups, with Glo-I activity being the lowest. Different glycation products and Glo-I activity were at their lowest and highest levels in group D (Thiamine & Niacin). Additionally, early glycation products (g-Alb and g-LDL) in group D (Niacin) were lower than in group D (Thiamine), while intermediate (MGO) and end glycation products (AGEs) were higher. The Glo-I activity in group D (Thiamine) was more in D (Niacin) *(P*<0.001*).*

**Figure 3:**
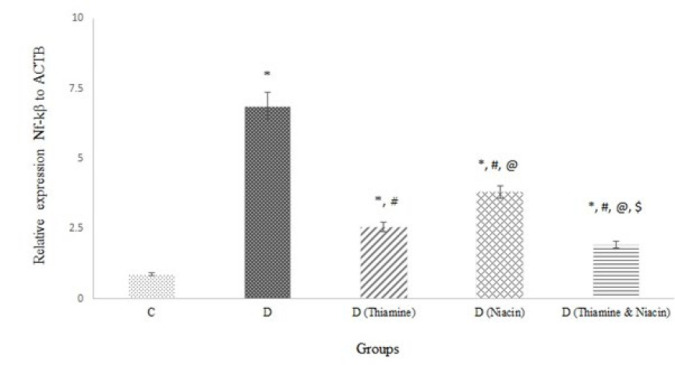
Comparison of relative Nuclear factor-kβ (NF-kβ) in thiamine and niacin-treated diabetic rats groups

Figure 3 displays the hepatic NF-kβ to ACTB gene expression ratio. The ratio in the D group was higher than in the other groups. The lowest ratio was found in the D (Thiamine & Niacin) group. Additionally, the ratio in D (Thiamine) was less than D (Niacin) *(P*<0.0*01).*

***Tube study***

Table 3 presents the impact of thiamine and niacin on the formation of diverse gly-oxidation products of Alb and LDL. The treatments reduced Alb glycation, LDL glycation, and oxidation products. The niacin tube had lower levels of cited products, while MGO and AGEs showed the opposite trend. 

## Discussion

The combination of thiamine and niacin had a more advantageous effect on macro and microvascular complications of diabetes than each vitamin alone. This is due to their higher reductive effect on oxidative stress, glycation, and inflammation. The vitamin combination improved glucose-lipid metabolism, insulin sensitivity, and renal function better by reducing hepatic NF-kβ signaling. Thiamine exhibited more anti-inflammatory activity than niacin in diabetic rats, while niacin has a stronger anti-oxidant action. Niacin’s anti-glycation effect was more prominent on early glycation products (g-Alb & g-LDL), while thiamine was more effective on intermediate (MGO) and end glycation (AGEs) products. 

Diabetes is characterized by persistent hyperglycemia, insulin dysfunction, and abnormal lipid metabolism that leads to diabetes complications. An increase in pro-inflammatory cytokines (such as IL-1β), various glycation products (especially MGO and AGEs), oxidized LDL, and FFA contribute to glycemia, insulin resistance, dyslipidemia, and obesity through activation of the hepatic NF-kβ pathway and reduction in GLUT4 gene expression (2, 19). Obesity exacerbates insulin resistance and diabetes by inducing oxidative stress. There is a direct correlation between reduction in body weight and the severity of insulin resistance (20). Individually and in combination, vitamins improved glycemia, insulin resistance, and dyslipidemia (Table 1) via a decrease in hepatic NF-kβ signaling (Figure 3) and its activators (Tables 1 & 2 and Figures 1 &2). The highest β cell activity (% β) and insulin sensitivity (% S) in D (Thiamine & Niacin) confirm the most advantageous effect of the vitamin combination on glycemia, insulin resistance, and dyslipidemia. Thiamine had a more beneficial impact than niacin on hyperglycemia and insulin resistance. The better effect of thiamine on glycemia and insulin resistance may be due to its higher activity in lowering the hepatic NF-kβ pathway, MGO accumulation or carbonyl stress, body weight, and FFAs levels, resulting in higher % β and lower HOMA. Thiamine had a greater reducing effect on MGO and AGE levels in both *in vitro* (Table 3) and *in vivo* (Table 2) conditions than niacin. Presumably, thiamine has more activity against carbonyl stress (MGO accumulation) through its higher di-carbonyls scavenging property and Glo-I induction. Niacin decreases FFAs due to superior lipolysis inhibition (21). This study reports for the first time the effect of the thiamine and niacin combination on glycemia, insulin resistance, % β, % S, NF-kβ expression, and its activators (IL-1β, glycation products, FFAs, and Ox-LDL). Moreover, the effect of the combination and niacin alone on mixed glycation products (*in vitro *and* in vivo*), Glo-I activity, % β, and % S has not been previously presented. Evidence suggests that thiamine complements in diabetic animals promote glycemia by increasing hexokinase activity and reducing glucose-6-phosphatase activity (22). Recent reports indicate that increasing dietary niacin intake promotes glucose homeostasis in adults over 40 (23). β-cells are highly sensitive to oxidative stress, which can interfere with insulin function and secretion (24). Therefore, anti-oxidant compounds are a perfect treatment against insulin resistance, diabetes, and diabetes-related complications. Thiamine and niacin alone and their combination with anti-oxidant activities improve the functions of β-cells and insulin. 

Raising IL-1β, NF-kβ signaling, insulin, insulin resistance, FFAs, MGO, and AGEs leads to dyslipidemia and vascular complications (25). Insulin resistance, inflammatory processes, and dyslipidemia have a reinforcing effect on each other (26). Thiamine deficiency causes vascular inflammation and dyslipidemia (27). Diabetes induction in rats increased their susceptibility to diabetes vascular complications, leading to dyslipidemia and elevating cardiovascular indices (LDL/HDL, MPO/HDL, and MPO/PON-1) (respectively, in Tables 1 and 2). Lower cardiovascular markers in the treated diabetic rats than in the untreated ones certify the vitamin’s protective effect against vascular complications in combination and alone. The vitamin combination had the best beneficial effect on lipid metabolism and the vascular system through the most reducing effect on IL-1β, NF-kβ signaling, insulin, insulin resistance, FFAs, MGO, and AGEs. There was no difference between thiamine and niacin effects on TC, HDL, LDL, and TG in diabetic rats, but FFA levels and all cardiovascular indices except LDL/HDL in thiamine-treated diabetic rats were lower. Thiamine has an essential function in lipid metabolism, and its deficiency via up-regulation of the hexosamine and NF-kβ pathways (28) causes dyslipidemia. Niacin is the most masterly therapeutic agent for dyslipidemia prevention and correction by elevating HDL-C and reducing TG, lipoprotein (a), and LDL levels (29). In this study, the vitamins, in combination and alone, corrected dyslipidemia and decreased the risk of vascular complications. For the first time, the effect of the vitamins in combination and alone on MPO/HDL and MPO/PON-1 was reported. FFAs, LDL glycation, and oxidation might have an axial role in the pathogenesis of atherosclerosis and nephropathy. Ox-LDL is a marker of oxidative stress, endothelial dysfunction (ED), and atherosclerosis (30). Reduced activity of paraoxonase 1 (PON-1), a high-density lipoprotein (HDL)-associated enzyme, has been implicated in the development of atherosclerosis. Under atherosclerotic conditions, MPO comrades with HDL and inhibits PON-1 activity via generating the oxidants hypochlorous acid and nitrogen dioxide (31). The advantageous effect of the treatments on lipid metabolism, PON-I, and MPO activities in the treated groups leads to a reduction of glycated and oxidized LDL products (Table 2) in the cited groups rather than untreated diabetic ones. The combination of the vitamins had the most protective effect on vascular and renal cells via the lowest glycated and oxidized LDL levels. Furthermore, niacin was a more potent inhibitor of LDL modifications than thiamine. The lowering effect of thiamine on TG and Chol levels in diabetic rats (22) and its effect on LDL glycation and oxidation (9) have been presented. A newly discovered corrective effect of niacin on dyslipidemia in type 2 diabetic patients (32) and the effect of niacin (33) on LDL oxidation were reported. The vitamin combination effect on LDL glycation and oxidation (early and end) and the niacin effect on LDL glycation in both *in vitro *and* in vivo* conditions have not been represented.

Reducing GFR (Table 1) and elevating TGF-β (Figure 1), Cr, and urinary protein excretion (Table 1) indicate renal dysfunction in rats following diabetes induction. The renal protective effect of the treatments is attributed to an improved impact on glycemia, insulin resistance, and dyslipidemia (Table 1), as well as a decrease in glycation, oxidative stress, and inflammation markers (Table 2 and Figures 1 & 2). Glycation products are significant contributors to diabetes complications through oxidative stress, inflammation, hyperglycemia, insulin resistance, and dyslipidemia enhancement (25). The kidneys and Glo-I play a crucial role in preventing diabetes complications by reducing glycation products (34). A decline in Glo-I activity and an increase in IL-1β levels contribute to the progression of diabetic nephropathy and atherosclerosis. Glo-I induction and IL-1β reduction have been identified as therapeutic targets for diabetic complications (35). Oxidative stress induction and elevated MPO activity lead to the development of diabetic nephropathy (36). Increased MPO activity contributes to cardiovascular complications and diabetic nephropathy through NF-kβ pathway activation and chlorotyrosine formation. Elevated NF-𝜅B signaling increases TGF-β1 levels as a key factor in diabetic nephropathy progression (37). Therefore, supporting the AGE-RAGE-NF𝜅B axis may be a strategic goal for improving diabetes vascular complications (38). In this study, oxidative stress markers (AOPP, MDA, and LDL oxidation products) and MPO activity increased in the diabetic group due to a decrease in the anti-oxidant profile (GSH level and CAT and PON-1 activities) (Table 2). The treatments reversed these changes in diabetic rats by reducing the formation of free radicals and inflammatory processes, increasing the anti-oxidant profile, and reducing IL-1β and MPO levels. Niacin exhibited greater anti-oxidant properties than thiamine in diabetic rats, but the combination produced treatment with enhanced anti-oxidant and anti-inflammatory properties. The coenzymes of niacin act as scavengers of free radicals and protect tissues against oxidative stress.

Beneficial effect of vitamins against vascular complications may be due to a reduction in TGF-1β (Figure 1), which decreases the AGE-RAGE-NF𝜅B axis. In diabetic rats under treatment with thiamine, niacin, and a combination of both higher GFR and lower TGF-β levels were observed compared to untreated rats, validating the protective effect of the treatments on kidney function. The combination of vitamins had the most significant impact on renal function, with thiamine showing more renoprotective activity than niacin. Thiamine’s superior effect on renal function was attributed to its higher Glo-I activity and lower levels of TGF-β, IL-1β, MPO, MGO, and AGEs. A lower KWI in thiamine-treated diabetic rats confirms a more beneficial effect on renal function. Compounds with high anti-oxidant properties are recommended for preventing or ameliorating diabetes vascular complications prevention or amelioration (39). A recent paper reported a high correlation between blood sugar, glycated hemoglobin, and serum thiamine quantities in diabetic patients. Additionally, thiamine supplementation may prevent diabetes-related vascular complications (40).

* Indicates significance of data comparing the control group with other groups (*P*<0.001)

# Indicates significance of data comparing the diabetic group with other groups (*P*<0.001)

@ Indicates significance of data comparing the thiamine-treated diabetic group with other groups (*P*<0.001)

$ Indicates significance of data comparing the niacin-treated diabetic group with other groups (*P*<0.001)

* Indicates significance of data comparing the control group with other groups (*P*<0.001)

# Indicates significance of data comparing the diabetic group with other groups (*P*<0.001)

@ Indicates significance of data comparing the thiamine-treated diabetic group with other groups (*P*<0.001)

$ Indicates significance of data comparing the niacin-treated diabetic group with other groups (*P*<0.001)

**Table 1:** The levels of metabolic profile and renal dysfunction parameters in the control (C), diabetic (D), and treated diabetic rats with thiamine, niacin, and their combination

Parameter	Groups				
	C	D	D (Thiamine)	D (Niacin)	D (Thiamine & Niacin)
FBS (mmol/l)	5.18 ± 0.30	19.10 ± 1.19^*^	8.27 ± 0.63^*, #^	9.33 ± 0.73^*, #, @^	7.49± 0.48 ^*, #, @, $^
Insulin (µU/ml)	15.01 ± 0.91	23.42± 1.36*	18.92 ± 1.29^ *, #^	17.44± 1.56^ *, #, @^	16.18± 0.53^ *, #, @, $^
HOMA	2.78 ± 0.18	19.67 ± 1.28*	6.95 ± 0.41^*, #^	7.23± 0.65^*, #, @^	5.38± 0.36^*, #, @, $^
%β	213.90 ± 14.03	22.71± 1.16*	68.10± 3.42^ *, #^	51.74 ± 1.69^ *, #, @^	72. 80 ± 3.22^ *, #, @, $^
%S	54.70 ± 2.37	15.83 ± 0.94*	37.10± 2.06^ *, #^	39.16 ± 1.94^ *, #^	44.00 ± 2.15^ *, #, @, $^
TG (mmol/l)	1.63 ± 0.08	3.27 ± 0.15*	2.36 ± 0.13^ *, #^	2.29± 0.09^ *,^^#^	1.99 ± 0.08^*, #, @, $^
TC (mmol/l)	2.36 ± 0.14	4.91± 0.20*	3.87 ± 0.16^*, #^	3.96 ± 0.17^*, #^	3.04 ± 0.13^ *, #, @, $^
HDL (mmol/l)	1.23 ± 0.07	1.01± 0.05^*^	1.70 ± 0.09^ *, #^	1.77± 0.11^ *, #^	1.39± 0.05^*, #, @, $^
LDL (mmol/l)	0.38 ± 0.03	2.41± 0.14^*^	1.09± 0.09^*, #^	1.13 ± 0.10^ *, #^	0.71 ± 0.07^ *, #, @, $^
LDL/HDL	0.30 ± 0.05	2.38 ± 0.17	0.64± 0.10^*, #^	0.63 ± 0.08^ *, #^	0.51 ± 0.05^*, #, @, $^
FFAs (µmol/l)	402.27 ± 24.86	711.04± 42.52^ *^	470.19 ± 27.74^ *, #^	451.82 ± 25.02^ *, #, @^	429.16 ± 24.14^ *, #, @, $^
Cr (µmol/l)	38.16± 3.01	99.75 ± 5.90 ^*^	59.22± 3.98^*, #^	71.42± 4.87^ *, #, @^	48.30 ± 3.25^ *, #, @, $^
PU (mg/dl/24 hr)	7.92 ± 0.56	301.23± 15.07^ *^	102.63 ± 7.10^ *, #^	135.58 ± 9.11^*, #, @^	69.38 ± 4.01^ *, #, @, $^
GFR (ml/min)	2.98± 0.24	1.39± 0.16^ *^	2.12 ± 0.20^*, #^	1.79 ± 0.15^ *, #, @^	2.41 ± 0.18^ *, #, @, $^
Body weight (g)	289.51 ± 15.72	373.68± 18.34^ *^	333.05 ± 16.22^ *, #^	349.31 ± 17.50^ *, #^	309.24± 16.40^*, #, @, $^
KWI (%)	0.79± 0.08	1.24± 0.16^ *^	0.86 ± 0.13^*, #^	1.02 ± 0.16^ *, #, @^	0.80 ± 0.21^ *, #, $^

* Indicates significance of data comparing the control group with other groups (*P*<0.001)

# Indicates significance of data comparing the diabetic group with other groups (*P*<0.001)

@ Indicates significance of data comparing the thiamine-treated diabetic group with other groups (*P*<0.001)

$ Indicates significance of data comparing the niacin-treated diabetic group with other groups (*P*<0.001)

FBG: fasting blood sugar; HOMA1: the original homeostasis model assessment of insulin resistance; HOMA2: the updated of HOMA model; %B: the percentage of β-cell function %S: the percentage of insulin sensitivity; TG: triglyceride; TC: total cholesterol; HDL: high density lipoprotein; LDL: low density lipoprotein; FFATs: free fatty acids; Cr: creatinine; PU: proteinuria in 24-hours; GFR: glomerular filtration rate; KWI: kidney weight index

* Indicates significance of data comparing the control group with other groups (*P*<0.001)

# Indicates significance of data comparing the diabetic group with other groups (*P*<0.001)

@ Indicates significance of data comparing the thiamine-treated diabetic group with other groups (*P*<0.001)

$ Indicates significance of data comparing the niacin-treated diabetic group with other groups (*P*<0.001)

**Table 2:** Comparison the levels of glycation, oxidative stress and inflammatory markers in the control (C), diabetic (D), and treated diabetic rats with thiamine, niacin, their combination

Parameter	Groups				
	C	D	D (Thiamine)	D (Niacin)	D (Thiamine & Niacin)
g-Alb (µmol/l)	83.91 ± 5.06	406.23 ± 24.10^*^	291.53 ± 17.36^ *, #^	260.11 ± 15.79^*, #, @^	184.32 ± 8.62^ *, #, @, $^
g-LDL (µmol/l)	24.04 ± 1.39	128.51 ± 6.84^*^	90.35 ± 4.92^ *, #^	73.01 ± 3.63^*, #, @^	59.84± 3.04^ *, #, @, $^
MGO (µmol/l)	9.02 ± 0.55	89.20 ±5.89 ^*^	57.61 ± 2.98^ *, #^	72.44 ± 4.16^ *, #, @^	39.01 ± 3.02^ *, #, @, $^
AGEs (FI, A.U)	46.41 ± 3.38	396.52 ± 25.04 ^*^	266.12± 17.01^ *, #^	307.33 ± 17.82^ *, #, @^	199.05 ± 12.81^ *, #, @, $^
AOPP (µmol/l)	9.82 ± 0.64	86.91 ± 5.83 ^*^	44.05 ± 2.70^ *, #^	37.44 ± 2.39^ *, #, @^	26.32± 1.93^ *, #, @, $^
MDA (µmol/l)	13.25 ± 0.68.	79.41 ± 4.21 ^*^	52.81± 2.61^ *, #^	46.64 ± 1.89^ *, #, @^	27.01 ± 1.64^ *, #, @, $^
CD (µmol/l)	10.59 ± 0.73	97.24 ± 6.03 ^*^	57.13 ± 2.82^ *, #^	43.25 ± 3.99^ *, #, @^	34.81 ± 1.95^ *, #, @, $^
FOPL (µmol/l)	215.61 ± 14.03	475.92 ± 21.50^ *^	365.04 ± 16.13^ *, #^	330.53 ± 15.51^*, #, @^	272.31 ± 1.60 ^*, #, @, $^
GSH (µmol/l)	199.01± 11.39	104.85 ± 5.28^*^	121.94± 7.01^*, #^	133.94± 8.56^*, #, @^	149.25± 9.38^*, #, @, $^
Glo-I (U/ml)	45.84± 2.01	21.06 ± 0.99 ^*^	33.95 ± 1.39^ *, #^	28.93 ± 1.44^ *, #, @^	37.50 ± 1.52 ^*, #, @, $^
PON-I (U/ml)	154.20± 9.01	51.09 ± 2.94*	90.33± 5.89^*, #^	103.42 ± 5.19 ^*, #, @^	125.03± 6.88 ^*, #, @, $^
CAT (U/ml)	136.92± 10.03	70.64 ± 4.11 ^*^	83.01± 6.16^ *, #^	105.71 ± 5.26^ *, #, @^	119.06 ± 7.15^*, #, @, $^
MPO (U/ml)	0.57± 0.04	5.85 ± 0.39^*^	1.06± 0.07^*, #^	1.44 ± 0.11 ^*, #, @^	0.89 ± 0.06 ^*, #, @, $^

* Indicates significance of data comparing the control group with other groups (*P*<0.001)

# Indicates significance of data comparing the diabetic group with other groups (*P*<0.001)

@ Indicates significance of data comparing the thiamine-treated diabetic group with other groups (*P*<0.001)

$ Indicates significance of data comparing the niacin-treated diabetic group with other groups (*P*<0.001)

g-Alb: glycated albumin; g-LDL: glycated LDL; MG: methylglyoxal; AGEs: advanced glycation end products; CD: conjugated dines; FOPL: fluorescent oxidation products of LDL; AOPP: advanced oxidation end products; MDA: malondialdehyde; GSH: reduced glutathione; IL-1β: interleukine-1β; Glo-I: glyoxalase-I; PON-I: paraoxonase-I; CAT: catalase; MPO: myeloperoxidase

**Table 3:** The effect of glucose (Glc) and the treatments (thiamine, niacin, and their combination) on the formation of various rat serum albumin (RSA) glycation products and LDL glycation and oxidation products in the test tube experiment

	Tube	Albumin-related glycation products		
		Glycated albumin (µmol/l)	Methylglyoxal (µmol/l)	AGEs (AU)
Albumin	Alb	81.53 ± 2.04	1.79 ± 0.16	37.20 ± 0. 90
	Alb+ Glc	902.07 ± 7.92	41.30 ± 0.59	431.37 ± 6.12
	Alb+ Glc + thiamine	234.57 ± 3.13	26.52 ± 0.19	228.84± 2.93
	Alb+ Glc + niacin	221.26. ± 2.88	34.96 ± 0.81	266.02 ± 3.91
	Alb+ Glc + (thiamine & niacin)	174.51± 2.36	16.67 ± 0.32	182.53 ± 1.97
LDL	Tube	LDL gly-oxidation products		
		Glycated LDL (µmol/l)	primary oxidation products (µmol/l)	final oxidation products (AU)
	LDL	23.76 ± 1.98	15.29 ± 0.14	13.66 ± 0. 86
	LDL+ Glc	341.08 ± 3.73	124.13 ± 0.64	479.05 ± 5.63
	LDL+ Glc + thiamine	234.57 ± 2.84	39.52 ± 0.25	228.84± 3.04
	LDL+ Glc + niacin	221.26. ± 3.01	27.96 ± 0.76	206.02 ± 4.16
	LDL+ Glc + (thiamine & niacin)	174.35± 1.87	18.47 ± 0.24	183.17 ± 2.01

Glc: glucose; RSA: rat serum albumin; LDL: low density lipoprotein

## Conclusion

The combination of thiamine and niacin had a more beneficial effect on macro and microvascular complications of diabetes than each vitamin alone. This is attributed to their higher anti-oxidant, anti-glycation, and anti-inflammatory properties. This vitamin combination positively impacted glucose-lipid metabolism, insulin sensitivity, and renal function due to its greater reducing effect on the NF-kβ pathway. However, further assessment of diabetic patients is needed to strengthen the evidence supporting these findings.

## Declaration

We have not used any AI tools or technologies to prepare this manuscript.
